# Assigning the right credit to the wrong action: compulsivity in the general population is associated with augmented outcome-irrelevant value-based learning

**DOI:** 10.1038/s41398-021-01642-x

**Published:** 2021-11-05

**Authors:** Nitzan Shahar, Tobias U. Hauser, Rani Moran, Michael Moutoussis, NSPN consortium, Edward Bullmore, Raymond J. Dolan, Ian Goodyer, Peter Fonagy, Peter Jones, Michael Moutoussis, Tobias Hauser, Sharon Neufeld, Rafael Romero-Garcia, Michelle St Clair, Petra Vértes, Kirstie Whitaker, Becky Inkster, Gita Prabhu, Cinly Ooi, Umar Toseeb, Barry Widmer, Junaid Bhatti, Laura Villis, Ayesha Alrumaithi, Sarah Birt, Aislinn Bowler, Kalia Cleridou, Hina Dadabhoy, Emma Davies, Ashlyn Firkins, Sian Granville, Elizabeth Harding, Alexandra Hopkins, Daniel Isaacs, Janchai King, Danae Kokorikou, Christina Maurice, Cleo McIntosh, Jessica Memarzia, Harriet Mills, Ciara O’Donnell, Sara Pantaleone, Jenny Scott, Beatrice Kiddle, Ela Polek, Pasco Fearon, John Suckling, Anne-Laura van Harmelen, Rogier Kievit, Sam Chamberlain, Edward T. Bullmore, Raymond J. Dolan

**Affiliations:** 1 Max Planck University College London Centre for Computational Psychiatry and Ageing Research, London, WC1B 5EH UK; 2 Wellcome Centre for Human Neuroimaging, University College London, London, WC1N 3BG UK; 3 Sagol School of Neuroscience, Tel Aviv University, Tel Aviv, Israel; 4 Psychology Department, Tel Aviv University, Tel Aviv, Israel; 5 Department of Psychiatry, University of Cambridge, Cambridge, UK; 6 Behavioural and Clinical Neuroscience Institute, University of Cambridge, Cambridge, UK; 7 Research Department of Clinical, Educational and Health Psychology, University College London, London, UK; 8 Medical Research Council Cognition and Brain Sciences Unit, University of Cambridge, Cambridge, UK

**Keywords:** Learning and memory, Human behaviour

## Abstract

Compulsive behavior is enacted under a belief that a specific act controls the likelihood of an undesired future event. Compulsive behaviors are widespread in the general population despite having no causal relationship with events they aspire to influence. In the current study, we tested whether there is an increased tendency to assign value to aspects of a task that do not predict an outcome (i.e., outcome-irrelevant learning) among individuals with compulsive tendencies. We studied 514 healthy individuals who completed self-report compulsivity, anxiety, depression, and schizotypal measurements, and a well-established reinforcement-learning task (i.e., the two-step task). As expected, we found a positive relationship between compulsivity and outcome-irrelevant learning. Specifically, individuals who reported having stronger compulsive tendencies (e.g., washing, checking, grooming) also tended to assign value to response keys and stimuli locations that did not predict an outcome. Controlling for overall goal-directed abilities and the co-occurrence of anxious, depressive, or schizotypal tendencies did not impact these associations. These findings indicate that outcome-irrelevant learning processes may contribute to the expression of compulsivity in a general population setting. We highlight the need for future research on the formation of non-veridical action−outcome associations as a factor related to the occurrence and maintenance of compulsive behavior.

## Introduction

To say that a reinforcement is contingent upon a response, may mean nothing more than that it follows the response. B.F. Skinner (1948) [1]

Compulsive, ritualistic behaviors are enacted to influence the likelihood that a certain event will occur [1]. These behaviors are seen in more than one-quarter of the general population [2, 3]. By definition, there is no causal relationship between compulsive behaviors and the likelihood of the event they aim to influence [4, 5]. Furthermore, in many instances, individuals have explicit knowledge that their compulsive actions are causally irrelevant [1]. Therefore, a fundamental unanswered question relates to what facilitates the formation of such action−outcome associations, given they do not exist in the external environment.

Outcome-irrelevant learning can be defined as a tendency to assign credit to actions that do not hold any causal association to an outcome [6]. Outcome-irrelevant learning was first observed in animals by Skinner, who found that pigeons acquire a ritualistic-like behavior when food pellets are presented at random time intervals [7]. He went on to note that movements enacted by chance, just before the appearance of a food pellet, were subsequently re-enacted at a higher frequency as if by doing so the pigeons could make the food pellet re-appear. For example, one of the pigeons learned to hop from its right to the left foot in a specific corner of the cage, despite this behavior having no causal influence on the future appearance of a food pellet. Whether such outcome-irrelevant learning, as observed in pigeons, bears any relation or significance to the expression of human compulsive behaviors is unknown.

Recently, we observed outcome-irrelevant learning in human subjects that manifests as a tendency to press a response key that was previously followed by a monetary gain, and a tendency to avoid it when it was followed by a loss, despite there being no actual causal relationship between the response key and an outcome [6]. Such outcome-irrelevant learning was observed even following extensive practice sessions (up to three sessions, and more the 500 trials), which should indicate to a participant that response keys were not predictive of an outcome [6]. On this basis, and bearing in mind the aforementioned studies on pigeons, we asked whether outcome-irrelevant learning might be a significant contributory process in the emergence of human compulsive behavior (i.e., behaviors that are not causally connected with the event they aim to influence). Thus, the main goal of the current study was to examine whether outcome-irrelevant learning is empirically related to compulsive tendencies in a community sample of human subjects.

Previous reinforcement-learning studies that have examined an association between value-based learning and compulsivity focused on goal-directed reasoning strategies (i.e., model-based control) [8–10]. These studies demonstrated a replicable (yet small) reduction in goal-directed reasoning strategies among individuals who scored high on compulsivity scales. Given that reduced model-based control is currently considered an inherent aspect of compulsive symptoms [8–10], it is of interest to ask whether outcome-irrelevant learning can extend our ability to predict the expressions of compulsivity, beyond that based on model-based control. Finally, some researchers have suggested that reduced model-based control is tightly coupled with an increased expression of habitual (model-free) behavior, such that a tendency to form and maintain rigid habits is considered to underlie compulsive behavior [11]. However, we note that empirical findings regarding increased habitual behavior in compulsive individuals provide mixed, and difficult to replicate, evidence [8, 12].

In the current study, we tested for an association between outcome-irrelevant learning and compulsive behavior in a healthy, general population sample, and to assess whether this association exists over and above other associated factors previously reported in the literature. We analyzed data from 514 individuals from a community-based longitudinal sample, comprising adolescent and young adult volunteers, living in Cambridgeshire and London, UK (Neuroscience in Psychiatry Network [13]). Participants completed self-report measures of obsessive, compulsive, anxious, depressive, and schizotypal tendencies, and performed a laboratory-based two-stage decision task [8, 14–18]. We first used latent score analysis to measure a latent factor of compulsivity and replicated Gillan et al.’s [9] findings by showing that compulsive behavior can be segregated from obsessive thinking using factor analysis, as well as replicated an association between reduced model-based abilities and compulsivity [8–10]. Importantly, we found that outcome-irrelevant learning positively relates to compulsivity, even after controlling for other clinical symptoms and the extent of expressed model-based control. Thus, our findings highlight a unique association between outcome-irrelevant learning and compulsive behavior in a healthy population of young people.

## Materials and methods

### Participants

We obtained data from a community-based longitudinal sample of adolescent and young adult volunteers living in Cambridgeshire and London, UK (Neuroscience in Psychiatry Network [13]). The study recruited participants from an age-sex-stratified sample, with equal numbers of males/females across five age groups: 14–15, 16–17, 18–19, 20–21, and 22–24.99 years. Participants completed up to three in-lab assessments, involving a structured psychiatric interview for DSM-V, clinical self-report measures, and task-based cognitive measures (median of 18 months between the first and last in-lab assessments). They further completed self-report measurements at three time points at home (median of 27.19 months between the first and third home-pack return; 5.76 months between the first in-lab and first home-pack return). Only participants who had been estimated on all measures (i.e., two-step task, and self-report measures) were included in further analysis. Participants who met the diagnostic criteria for a psychiatric disorder were excluded (*N* = 31; see Supplementary Information), resulting in a total of 514 individuals (females/males = 255/259; mean age at first assessment = 18.39; 6.23% Asian, 4.47% Black, 6.23% Mixed, 76.46% White, 6.61% other). The study was carried out in accordance with the Declaration of Helsinki and Good Clinical Practice guidelines. Ethical approval was granted by the Cambridge Central Research Ethics Committee (12/EE/0250), and all participants gave their informed consent to take part in the study.

### Self-reported symptoms

Self-report ratings regarding symptoms of compulsive, obsessive, anxious, depressive, and schizotypal tendencies were obtained by asking participants to complete the following scales:

- *Obsessive-Compulsive Inventory-Revised* (OCI-R) [19, 20]—18-items, divided into six subscales: Washing, Checking, Ordering, Counting, Hoarding, and Obsessions.
- *Padua Inventory-Washington State University Revision* (PI-WSUR) [21, 22]—39 items, divided into five subscales: Thoughts about harm, Impulses to harm, Washing, Checking, and Grooming.
- *Short Leyton Obsessional Inventory* (LOI) [23]—11 items, totaled to create a single sum score.
- *Mood and Feelings Questionnaire* (MFQ) [13, 24]—33 items, totaled to create a single sum score.
- *Revised Children’s Manifest Anxiety Scale* (RCMAS) [13, 24, 25]—37 items, divided into three subscales: Physiological-anxiety, Worry, Social-anxiety.
- *Schizotypal Personality Questionnaire* (SPQ) [26]—74 items, divided into nine subscales: Ideas of reference, Odd behavior, Constricted affect, Odd speech, Excessive social-anxiety, No close friends, Suspiciousness, Perceptual experiences, Magical-thinking.

**Fig. 1:**
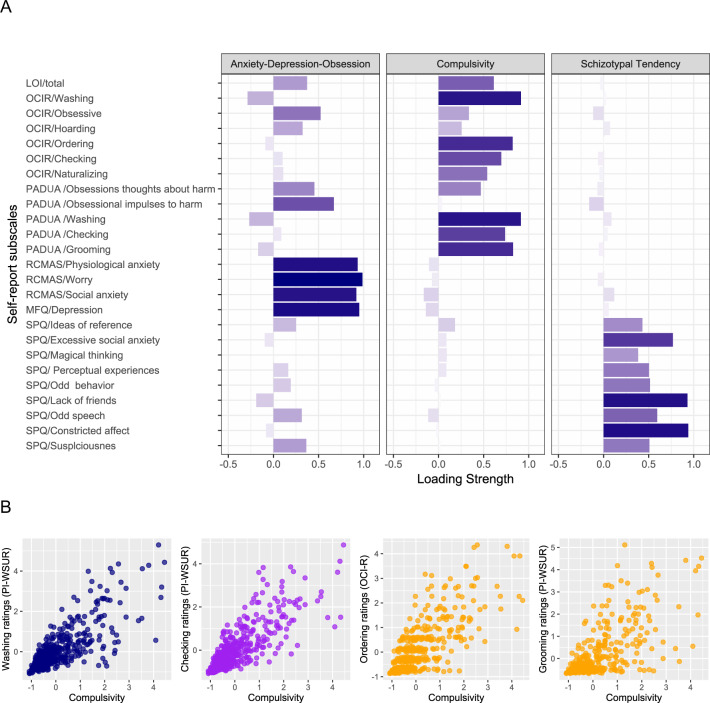
The three factors revealed from a dimension reduction analysis performed on self-report estimates. **A** Factor loadings showed that obsessional thinking loaded primarily on the first factor, along with depression, anxiety, and worry subscales. The compulsive behavior subscale primarily loaded on the second factor, and schizotypal tendencies predominantly loaded on the third factor. **B** Illustration of the association between the compulsive factor and individual items from the self-report measures. The *x* axis on all four scatter plots indicates the latent score for each individual on the compulsivity factor. The *y* axis illustrates the subscale score of a single estimate including (from left to right): (I) Compulsive washing (the average rating across the ten items of the washing subscale in the PI-WSUR questionnaire; an example item is, “I wash my hands more often and longer than necessary.”). (II) Compulsive checking (the average rating across the ten items of the checking subscale in the PI-WSUR questionnaire; an example item is, “I tend to keep on checking things more often than necessary”). (III) Compulsive ordering behavior (the average rating across the three items of the ordering subscale in the OCI-R questionnaire; an example item is, “I get upset if objects are not arranged properly”). (IV) Compulsive dressing/grooming (the average rating across the three items of the grooming subscale in the PI-WSUR questionnaire; an example item is, “I feel obliged to follow a particular order in dressing, undressing, and washing myself”).

Overall, the six questionnaires (i.e., OCI-R, PI-WSUR, LOI, MFQ, RCMAS, and SPQ) resulted in 25 subscales (see Supplementary Table S1). Using linear regression, we controlled for age, gender, and repeated assessments for each of the 25 subscales, followed by a dimension reduction analysis (for a single time point analysis controlling for change over time in symptoms; for information on how these estimates were controlled, see Supplementary Information). Performing a dimension reduction analysis was important for several reasons; first, a previous study demonstrated that dimension reduction can disentangle compulsive from obsessive tendencies [9]; second, dimension reduction is useful in reducing multiple comparisons [8, 9]; and third, dimension reduction allows for an estimation of latent factors, known to be more reliable than single estimates [27]. Our dimension reduction analysis involved a principal component analysis (a ‘promax’ rotation was used to allow for non-orthogonal components while avoiding factor score indeterminacy, which is characteristic of factor analysis; see Supplementary Information). Using a scree plot analysis (see Supplementary Information and Supplementary Fig. S1), we observed that three factors best explained the data (58% explained variance). After examining subscale loadings on each of the factors (Fig. 1), we labeled the first factor ‘anxiety−depression−obsession’, the second ‘compulsivity’ and the third ‘schizotypal tendency’ (22%, 21% and 16% explained variance for each factor, respectively). Importantly, similar to findings from a recent study by Gillan et al. [9], dimension reduction enabled us to segregate obsessive from compulsive tendencies. Overall, this analysis resulted in three factors, with each of the 514 individuals having a single loading score on each factor (compared with 25 estimates for each individual before dimension reduction). These three factors were used in a subsequent regression analyses, as presented below.

### Reinforcement learning estimates

#### Two-step task

**Fig. 2:**
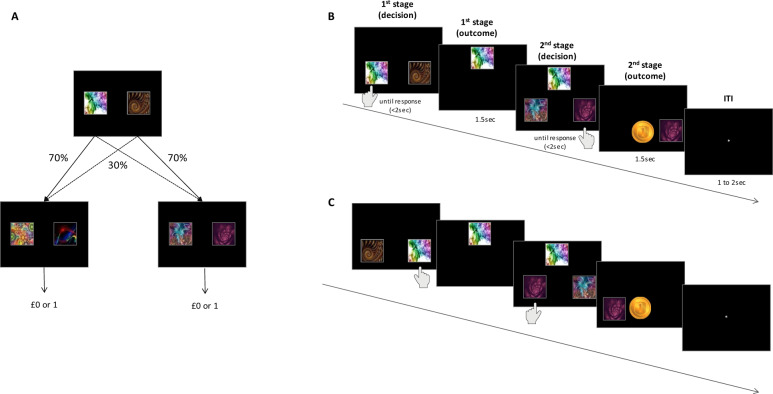
Two-step task illustration. **A** Participants navigated between the two stages of the task in order to reap rewards. The second stage included two pairs of fractal images, which led probabilistically to a reward. To attain these rewards, participants made choices during the first stage, which probabilistically determined the fractals presented during the second stage. **B** Illustration of trial sequences, showing a choice made in the first stage, followed by feedback, and a second-stage selection that was followed by a reward (1 play pound). **C** A fractal to response key pairing was allocated randomly in each trial. Panel (**C**) illustrates a trial sequence, in which the same fractals were selected as in panel (**B**), but now with different effectors. Although fractal identity predicted relevant outcomes (second-stage fractals, and reward), the position of the fractal and the response key used to report a selection were always outcome-irrelevant. Outcome-irrelevant learning was inferred from a participant’s tendency to assign value to response keys despite their irrelevance to any individual decision (see Fig. 3 for outcome-irrelevant estimate plots). Model-based control was estimated as the ability to select a first-stage action based on the task’s transition probability and subjective action values of second-stage fractals [14, 31] (see Supplementary Fig. S3 for model-based estimate plots).

To obtain individual measures of outcome-irrelevant learning and model-based control, participants completed a two-step reinforcement-learning task [14, 18, 28]. In this task, players were asked to make decisions in order to maximize monetary gains (play pounds). Each trial included two stages, in which participants made a choice between two fractals (see Fig. 2A). The fractals in the first stage led probabilistically to one of the two second-stage pairs; fractals in the second stage led probabilistically to receipt of a reward (£0 or £1). Each fractal was randomly assigned in each trial and stage to appear on the left or right side of the screen. Participants were instructed to select a fractal by pressing the corresponding left or right response key (see Fig. 2B). Importantly, only fractals, but not their arbitrary and varying affiliated response keys, predicted outcomes—the fractal position on the screen, and the effector participants used to select the fractal were randomly assigned by the computer. Subjects’ choices and reaction times enabled us to estimate outcome-irrelevant learning and model-based control, as described further below.

#### Outcome-irrelevant learning

**Fig. 3:**
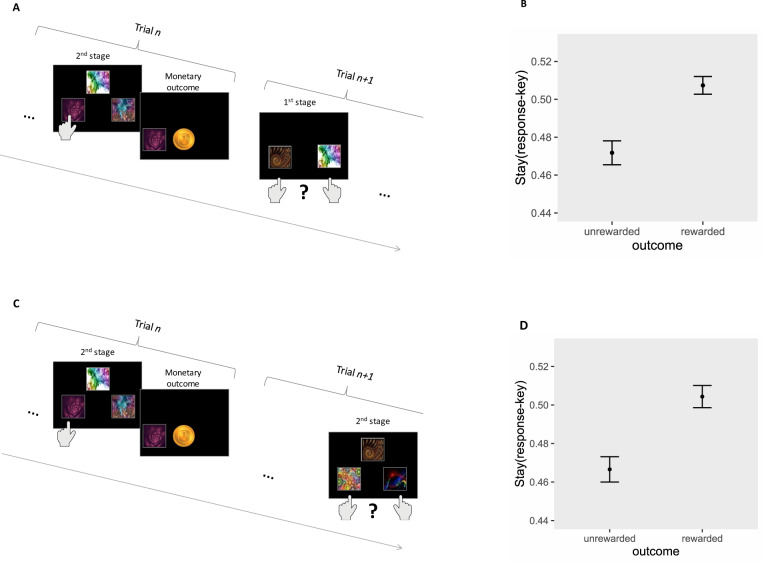
Outcome-irrelevant learning. The figure illustrates two sequential trial analyses (previously reported in Shahar et al.), demonstrating outcome-irrelevant value learning. These analyses examined a tendency to repeat a response key selection from trial *n* to trial *n* + 1, as a function of reward. **A**, **B** First-stage score—In this analysis, we show the influence of reward delivery on a tendency to re-select a response key during the first stage of the *n* + 1 trial, which was previously selected in the second stage of the *n* trial. For example, if the individual selected a fractal with a left response key press in the second stage of trial *n*, the left response key is more/less likely to be selected in the first stage of the following trial as a function of the reward/unrewarded outcome in trial *n*, respectively, as shown in panel (**B**). **C**, **D** Second-stage score I—In this analysis, we demonstrate the influence of reward delivery on a tendency to re-select a response key in the second stage of the *n* + 1 trial, which was previously selected during the second stage of the *n* trial. This analysis included only trials in which a different pair of fractals was offered in the *n* and *n* + *1* trial. For example, if the individual selected a fractal with a left response key press in the second stage of trial *n*, the left response key is more/less likely to be selected in the second stage of the following trial as a function of reward/unrewarded outcome in trial *n*, as shown in panel (**D**) (for second-stage score II, see Supplementary Iinformation).

We operationalized outcome-irrelevant learning as a disposition to assign value to a task representation that is not predictive of an outcome (see Fig. 3). In the current task, fractals were randomly assigned in each trial to appear on either the left or right side of the screen, and participants pressed a corresponding left or right response key to select a fractal (see Fig. 2B). Fractal identity alone, but not their response keys, predicted reward. This task feature was introduced to participants by means of both instruction and practice. We started by re-fitting a computational model that allows estimation of individuals’ tendency to assign value to the response key, despite it being outcome-irrelevant [6]. Specifically, Shahar et al. [6] previously reported findings from a comprehensive model comparison using the same data and showed that a tendency to assign and follow outcome-irrelevant representations is captured by two computational model parameters: a decision-weight, reflecting the integration of such information during the decision phase (*w*_outcome-irrelevant_; see Supplementary Information, Eqs. 6 and 7 and Table 1) and a learning-rate parameter, reflecting the updating of outcome-irrelevant representations (*α*_outcome-irrelevant_; see Supplementary Information, Eqs. 3 and 4 and Table 1). After fitting the computational model and estimating *w*_outcome-irrelevant_ and *α*_outcome-irrelevant_, we further estimated three independent sequential trial scores previously found to be closely related to these two outcome-irrelevant computational parameters [6]. This was implemented because the use of both model parameters, and closely related model-agnostic scores, have been shown to increase estimator reliability [17]. Furthermore, a non-computational-minded reader will find it easier to understand outcome-irrelevant learning by considering these model-agnostic estimates, which directly reflect outcome-irrelevant learning.

- *First-stage score* (see Fig. 3A)—calculated as the effect of a trial *n* reward (unrewarded vs. rewarded) on the probability that a response key selection made in the second stage of the *n* trial will be repeated in the first stage of the *n* + 1 trial.
- *Second-stage score I* (Fig. 3B)—calculated as the effect of a trial *n* reward (unrewarded vs. rewarded) on the probability that the response key selection made in the second stage of the *n* trial will be repeated in the second stage of the *n* + 1 trial. This score was calculated using trials in which the individual reached a different second stage in trial *n* + 1 compared with trial *n*, ensuring that the effect would not be influenced by value-learning regarding the relevant fractals.
- *Second-stage score II* (see Supplementary Fig. S2)—calculated as an interaction of a trial *n* reward (unrewarded vs. rewarded) and fractal to response key pairing (same vs. different) on the probability that the same fractal will be selected again in trial *n* + 1. This score was calculated using responses from two consecutive trials, in which an individual was offered the same pair of fractals at the second stage. A similar effect of reward across pairing (no interaction) indicates no credit assignment to the response key, whereas a positive interaction indicates an assignment of value to the response key.

**Table 1:** Sample characteristics and descriptive data per time point.

	Baseline	Follow-up 1	Follow-up 2	Across time points
Sample characteristics				
*N*	514	48	514	
Gender (m/f)	259/255	24/24	259/255	
Age	18.81 (2.96)	19.30 (2.87)	20.27 (2.98)	
Outcome-irrelevant learning				
*w*_outcome-irrelevant_				0.23 (0.09)
*α*_outcome-irrelevant_				0.29 (0.23)
First-stage score	0.03 (0.12)	0.04 (0.11)	0.04 (0.10)	
Second-stage score I	0.04 (0.15)	0.04 (0.14)	0.03 (0.12)	
Second-stage score II	0.15 (0.27)	0.12 (0.24)	0.16 (0.22)	
Model-based control				
*w*_model-based_				0.38 (0.20)
First-stage score	0.10 (0.25)	0.08 0(0.23)	0.12 (0.20)	
Second-stage score (ms)	120 (110)	110 (90)	130 (100)	

Outcome-irrelevant learning: *w*_outcome-irrelevant_ reflects the weight of the response key cached value on the individual’s trial-by-trial decisions (units are arbitrary and should be interpreted in terms of being negative, zero, or positive; see Supplementary Information, Eqs. 6 and 7), estimated using computational modeling across all three time points. *α*_outcome-irrelevant_ is the learning rate for the response key cached value (range is between 0 and 1; see Supplementary Information, Eqs. 3 and 4), estimated across all three time points. First-stage and second-stage score I estimates are depicted as unstandardized regression coefficients, representing the effect of outcome in the previous trial (rewarded vs. unrewarded) on the probability of making the same response key choice (see Fig. 3). Second-stage score II shows the unstandardized regression coefficients of the previous outcome × mapping interaction estimate on the probability of making the same response key choice (Supplementary Fig. S1). Model-based control (*w*_model-based_) reflects the weight of model-based strategies on an individual’s first-stage choices (units are arbitrary and should be interpreted in terms of being negative, zero, or positive; see Supplementary Information, Eq. 6). The first-stage score shows the unstandardized regression coefficients of the previous reward × previous transition interaction effect on the probability that individuals will repeat their first-stage fractal choice (see Supplementary Fig. S2). Second-stage score II reflects the difference in reaction time for second-stage choices after a rare compared to a common transition (see Supplementary Fig. S2).

Summary statistics for the five outcome-irrelevant learning estimates can be found in Table 1. Correlations between the five outcome-irrelevant learning scores were all positive, as expected. Pearson coefficients for the correlations of *w*_outcome-irrelevant_ with first-stage and second-stage I and II scores were 0.12, 0.11, and 0.15, respectively (*p* < 0.05). Correlations of *α*_outcome-irrelevant_ with first-stage and second-stage I and II scores were 0.35, 0.45, and 0.41, respectively (*p* < 0.001). After controlling for age, gender, and repeated assessments using linear regression (see Supplementary Information for details regarding this analysis), we transformed the five estimates to standardized *z*-scores and averaged across these to obtain a single compound score reflecting outcome-irrelevant learning (for a single time point analysis controlling for change over time in symptoms, and information regarding how these estimates were controlled, see Supplementary Information).

A number of issues regarding outcome-irrelevant learning estimation need consideration. First, this type of learning was observed despite extensive task experience [7]. In fact, we found that even after three in-lab sessions and more than 500 trials, outcome-irrelevant learning was still evident in an individual’s behavior and, if anything, tended to increase towards the end of each session (see analysis in Shahar et al. [7]). Second, a study by Feher da Silva and Hare [29], which used a cover story two-step task version to ensure that instructions were clear, provided additional support for our findings. A re-analysis of the two-step task data from Feher da Silva and Hare (2020) showed that the use of an explicit task cover story did not eliminate outcome-irrelevant learning (see Supplementary Information). Thus, outcome-irrelevant learning was observed despite individuals receiving clear and reliable instructions that response keys do not predict an outcome per se. Finally, recall that outcome-irrelevant learning, in the current task, refers solely to a tendency to repeat a response key selection as a function of reward. Our computational model also included two free parameters capturing key perseveration (a tendency to repeat the previously selected response key regardless of reward) and key bias (a tendency to prefer right or left response keys regardless of task history, e.g., due to hand dominancy); both can influence response key selection independent of reward delivery (see Supplementary Information). Key perseveration and key bias were not included as outcome-irrelevant learning estimates as they do not reflect value assignment.

#### Model-based control

Model-based strategies are an expression of goal-directed control, which utilize explicit knowledge about the transition structure of the environment in order to inform the best option choices [9, 14, 30]. A model-based system calculates action values by prospectively examining a chain of outcomes that are expected to follow a specific action or set of actions. In the current two-step task, model-based control assigns value to first-stage visual stimuli (i.e., fractals) by calculating which of the two first-stage fractals is most likely to lead to the best second-stage fractal. We assessed model-based control using a well-described computational parameter, which calculates a weighting for the relative influence of model-based strategies on decision-making (*w*_model-based_; see Supplementary Information, Eq. 6 and Table 1). We further estimated two independent sequential trial scores, which were previously found to be directly associated with the computational *w*_model-based_ parameter [30]. The aggregation of these three estimates was shown to provide a reliable model-based control latent variable.

- First-stage score (see Supplementary Fig. S3)—The interaction effect of transition (common vs. rare) and outcome (rewarded vs. unrewarded) from the previous trial on the probability of repeating a first-stage choice on the next trial. For the non-computational reader, a brief explanation is called for as to why this interaction is considered to reflect model-based control, that is, an ability to make first-stage decisions based on transition probabilities and subjective second-stage values. Assume your choices in trial *n* led to a reward. When making a first-stage choice in the *n* + 1 trial, an individual using a model-based strategy will take the transition structure into account. If the previous trial included a common transition, this individual will stay with the same first-stage choice, as this provides the best chance of reaching the same previously rewarded second-stage fractal. However, if the previous trial included a rare transition, then an individual who relies on a model-based strategy, should switch to the alternate first-stage choice, since this has a greater probability of leading to the same second-stage fractals which were rewarded in the previous trial. Therefore, a higher transition × reward interaction score is considered indicative of a model-based strategy [14, 30].
- Second-stage score (see Supplementary Fig. S3)—An individual who deploys model-based strategies in the first stage also demonstrates faster reaction time cost at the second stage [16, 30]. A reaction time cost is calculated as the difference between the mean reaction time in the second stage after a rare vs. common transition, in which a larger difference (i.e., larger MB-II scores) indicates greater model-based involvement.

Summary statistics for the three model-based estimates of interest can be found in Table 1. Correlations between the three model-based scores were positive, as expected. The Pearson correlation was 0.55 between the first- and second-stage scores, 0.51 between the *w*_1_ parameter and the first-stage score, and 0.37 between the *w*_model-based_ parameter and the second-stage score (all *p* values were <0.001). We controlled for age, gender, and repeated assessments for each estimate, then transformed the three estimates to standardized *z*-scores and averaged them to obtain a single compound score reflecting model-based control (for a single time point analysis controlling for change over time in symptoms, and information regarding how these estimates were controlled, see Supplementary Information).

A few caveats regarding model-based control estimates need to be acknowledged. First, as studies have raised concerns regarding estimates derived from first-stage scores [18, 28, 29, 31], we took several steps to ensure the integrity of our estimates. These steps included: (1) using both first-stage choices and second-stage reaction time estimates [30] and (2) adhering to recommendations of Akam et al. of a need to control for choice accuracy at the first-stage choice, as the latter improves the validity of first-stage model-based estimates (see Supplementary Information). To further ensure that we had good reliability estimates, we followed hierarchical model fitting procedures [32], which resulted in behavioral estimates of ~0.8 test−retest reliability or more in the two-step task [30, 32]. In addition, in line with previous literature suggesting that aggregating both measures of choice and reaction times [16, 30] into a single compound score greatly improves the psychometric properties of the estimates, we also performed this calculation. Finally, we did not include model-free estimates in our main analysis since previous studies failed to show a relationship between these estimates and compulsivity (see Supplementary Information for the model-free estimates).

## Results

**Fig. 4:**
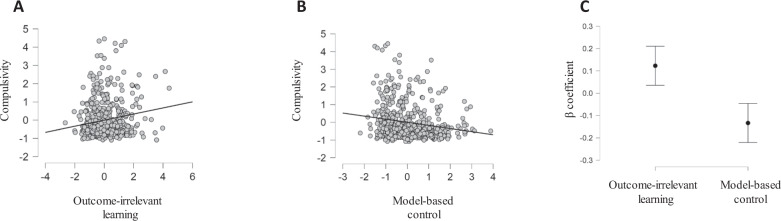
Association of outcome-irrelevant learning and model-based control with compulsivity. **A** Scatterplot showing the association between outcome-irrelevant learning and compulsivity. **B** Scatterplot showing the association between model-based control and compulsivity. **C** Posterior mean coefficients with 95% credible intervals taken from a Bayesian regression analysis exploring the effects of outcome-irrelevant learning and model-based control on compulsivity. Overall, the results show that outcome-irrelevant learning is positively related to compulsivity, even when controlling for the impact of model-based control (note that estimates are presented as standardized scores. Outcome-irrelevant learning estimates reflect the compound scores across five closely related task-based estimates, model-based control estimates reflect the compound scores across three closely related task-based estimates, and compulsivity reflects a factor that mainly loaded on self-report items of washing, checking, ordering, and grooming).

Our main question was whether outcome-irrelevant learning is associated with compulsivity. Thus, we examined the correlation between latent compulsivity factor scores and outcome-irrelevant learning estimates (see Fig. 3). Outcome-irrelevant learning showed a positive correlation with compulsivity, confirming our main hypothesis (*r* = 0.17, CI_95%_: 0.08–0.25, BF_10_ = 140.47 in support of H1, see Fig. 4A; also for posterior distribution plot and prior robustness check, see Supplementary Fig. S4). This result indicates that individuals who display a tendency to assign credit to task elements that are not related to an outcome are also those who report higher compulsive tendencies. Note that while this effect is considered quite small based on recent individual differences guidelines [33] (~3% explained variance), the effect size we report is very similar in magnitude to those reported in previous studies examining an association between value-learning and compulsivity [8–10].

Next, we replicate a finding reported in previous studies, where we find a negative correlation between compulsivity and model-based abilities [8–10] (*r* = −0.18, CI_95%_: −0.26 to −0.09, BF_10_ = 272.74 in support of H1, see Fig. 4B; also for posterior distribution plot and prior robustness check, see Supplementary Fig. S4). Thus, we found model-based control was negatively correlated with outcome-irrelevant learning (*r* = −0.29, CI_95%_: −0.37 to −0.21, BF_10_ = 8.84 × 10^8^; see Supplementary Fig. S5). This raises the question as to whether outcome-irrelevant learning improves our ability to explain individual variability in compulsivity estimates, beyond a well-established association between model-based control and compulsivity [8–10].

To examine an association between outcome-irrelevant learning and compulsivity, while controlling for model-based abilities, we next conducted a multiple Bayesian linear regression. In this analysis, we tested the effects of outcome-irrelevant learning and model-based abilities, as well as their interaction, on compulsivity. Following recent guidelines for Bayesian linear regression [34], we first conducted model comparison, followed by an examination of the parameters posterior distributions for the winning model (for a null hypothesis testing table with *p* values, see Supplementary Table S2). We tested five nested models including (a) a null model (with an intercept predicting compulsivity), (b) outcome-irrelevant learning as a single predictor of compulsivity, (c) model-based control as a single predictor of compulsivity, (d) both outcome-irrelevant learning and model-based control predicting compulsivity, and finally (e) the impact of the two main effects, and their interaction, as predictors of compulsivity. We found that the data were most likely under a model containing both outcome-irrelevant learning and model-based control as predictors of compulsivity, with no interaction (i.e., winning model; *R*^2^ = 4.5%). The results indicated that the data are 1435.92 times more likely under the winning model compared to the null model, 6.15 times more likely compared to a model with only model-based control as a predictor, 11.87 times more likely compared to a model with only outcome-irrelevant learning as a predictor of compulsivity, and 2.69 times more likely compared to a model with both outcome-irrelevant learning, model-based control and their interaction as predictors of compulsivity. Examining the posterior parameter distributions for the winning model showed that higher outcome-irrelevant learning (coefficient posterior mean = 0.12, CI_95%_ = 0.04−0.21) and lower model-based abilities (coefficient posterior mean = −0.13, CI_95%_ = −0.22 to −0.06) predicted higher compulsivity estimates (see Fig. 4C). These results were robust across a range of priors (for prior robustness checks, see Supplementary Information). Overall, this result supports a proposal that outcome-irrelevant learning predicts compulsivity after controlling for model-based abilities.

Our latent factor of compulsivity was obtained using a non-orthogonal rotation, as this allowed us to deal with factor indeterminacy and provided us with an easier way to interpret factor estimates. However, it also meant that clinical factors were expected to be correlated (see Supplementary Fig. S6). We repeated the same Bayesian linear regression described above, with the only difference being that anxious, depressive, obsessive, and schizotypal tendencies were included as additional null predictors across all models. We found that the winning model was one where outcome-irrelevant learning was a single task-based predictor (see Supplementary Table S3 for Bayes factors and Supplementary Table S4 for the null hypothesis testing analysis with *p* values). The reason model-based control was not included in the winning model is likely to reflect the fact that model-based control was more highly correlated with the two additional clinical factors, and therefore less specific to predicting compulsivity (see Supplementary Fig. S6 for a correlation matrix between the factors).

Finally, one concern in our analysis comes from the use of individual random effect coefficients for subsequent analyses, a procedure that can underestimate variances and overestimate the covariance [35]. To rule out the influence of the latter we repeated our analysis with outcome-irrelevant learning and model-based scores that were estimated individually (as opposed to hierarchically, with random effects). This analysis led to the same conclusions (see Supplementary Information).

Thus, our main finding is a positive association between outcome-irrelevant learning and compulsivity. Despite the small effect size (~3% explained variance), this association remained significant even after controlling for model-based control, and the co-occurrence of obsessive, anxious, depressive, and schizotypal tendencies.

## Discussion

Compulsive rituals are often performed under the belief that they alter the probability of the occurrence of some future event [1, 36]. Here, we demonstrate that a tendency to form action−outcome associations, that do not exist in the external environment (i.e., outcome-irrelevant learning), is associated with higher levels of compulsive symptoms in the general population. Albeit small, the positive association between outcome-irrelevant learning and compulsivity remained when accounting for model-based control, as well as anxious, depressive, and schizotypal tendencies.

A remarkable element of outcome-irrelevant learning estimates is that they are expressed across outcome-relevant features of the task (i.e., fractals, states, and stages) [6, 37]. This suggests that compulsive rituals might, in part, represent response-outcome tendencies that are divorced from any influence of decision-relevant stimuli [6]. For example, think of a bowler who has just thrown a ball and is now moving her shoulders from right to left, as if she is trying to control the course of the ball [7]. An action (e.g., twisting the shoulders) might then be reinforced by the outcome (e.g., a high score), irrespective of any outcome-relevant aspects (e.g., feeling the ball in one’s hand or visually examining the bowling lane before throwing the ball). This means that, at some subjective level, shoulder twisting becomes associated with better bowling outcomes, and on this basis might come to be perceived as having a ‘magical’ influence on the ball’s trajectory [1, 7].

A prominent observation in the reinforcement-learning literature regarding compulsive behavior is that individuals with high compulsive tendencies show reduced model-based control [8, 9, 11, 38, 39], a finding also supported by our current study. Importantly, we found that an association between outcome-irrelevant learning and compulsivity remains even after controlling for model-based abilities. Our findings further suggest that outcome-irrelevant learning was slightly less associated with other psychiatric symptoms (i.e., anxiety, depression, and schizotypal tendencies) compared to model-based abilities. When we controlled more strictly for these additional clinical symptoms, we found that the best model to predict compulsivity was the one with outcome-irrelevant learning as a single predictor, without benefits for adding model-based abilities as an additional predictor. Therefore, it might be that model-based abilities are more related to general psychopathology, while outcome-irrelevant learning is more directly associated with compulsivity. However, dedicated studies are required to address this assumption. We further suspect given these results that future studies involving sub-clinical screening and/or assessments might yield improved prediction accuracy for compulsivity with respect to outcome-irrelevant learning estimates. However, to accomplish the goal of using task-based estimates for sub-clinical screening, further studies will need to be cognizant that an empirical association between task-based estimates and compulsivity tends to be small [8–10].

Another related issue is that the current study did not address possible theoretical reasons as to why model-based control was negatively associated with outcome-irrelevant learning, and we suggest this as a useful focus for future investigation. Interestingly, Moran et al. argued that a cognitive map (or model) guides credit assignment [40–43], specifically the attribution of relevant rewards to a preceding causal action. By extension, we can speculate that a cognitive model of the environment includes a representation of which aspects of an action (e.g., visual or motor) are relevant to a task outcome, thus directing credit assignment to relevant aspects and filtering out any credit assignment to non-relevant aspects. Future studies might usefully examine whether a direct manipulation of model-based resources impacts upon outcome-irrelevant learning, which might, in turn, influence the expression of compulsive tendencies.

Our results have relevance for the interpretation of findings from value-based neuroimaging studies on compulsive individuals. Specifically, a blunted neural response to a reward has been reported in compulsive individuals, with areas such as the nucleus accumbens showing reduced reward anticipation encoding [44]. In contrast, other studies have reported increased reward prediction error signals among compulsive individuals [45, 46]. Our findings imply a much more complex expression of reinforcement learning among highly compulsive individuals. Thus, studies addressing reward-related neural responses among highly compulsive individuals might endeavor to disentangle outcome-relevant from outcome-irrelevant reward-related responses. We acknowledge that our results speak to tendencies in the general population and any generalization to a clinical population, such as those with obsessive−compulsive disorder, should be made with caution pending further evidence [9].

One limitation to the current study is that we cannot determine a direction of causality using regression analysis alone [47, 48]. Many studies tend to implicitly infer that task-behavior reflects cognitive processes that underlie compulsive behavior [11, 38, 39]. For example, it might be the case that outcome-irrelevant learning reflects a general tendency of the cognitive system to assign credit and form non-veridical action-outcome associations, which then leads to increased compulsive behavior. This suggests, much like Skinner’s superstitious pigeons [7], that some actions that are enacted prior to a meaningful outcome (or internal imagery of such an outcome [1]) can be perceived as causally related to that outcome. However, studies place less of an emphasis on the fact that task-based performance might be seen as reflecting a set of symptoms, rather than representing an underlying mechanism. According to this view, a latent tendency towards compulsive action leads to a specific behavior in our task, such that participants were less able to think and act in a model-based manner and were more prone to repeat a response key selection after a reward. Therefore, both reduced model-based behavior and increased outcome-irrelevant learning might reflect underlying causal factors in the genesis of compulsive tendencies. Yet, equally plausible is the possibility that reduced model-based behavior and increased outcome-irrelevant learning are themselves consequences of compulsive tendencies. Only a rigorous manipulation of model-based control and outcome-irrelevant learning will ultimately enable us to determine which explanation is more likely [48].

Another potential limitation relates to a suggestion that the deployment of model-based strategies, such as in Daw et al.’s task version, do not necessarily lead to higher gains. This means model-based estimates such as ours might underestimate an individual’s true ability, as participants might not have been motivated to deploy model-based strategies [18, 31]. This might explain the small observed overall effect in our study, and indicates future studies that encourage the use of model-based abilities will be informative. However, notwithstanding a potential underestimation of the true association between model-based abilities and compulsivity, this is less likely the case when it comes to estimates of outcome-irrelevant learning, the main focus of the current study. Outcome-irrelevant learning in the two-step task leads, by definition, to lower pay-offs, as these aspects change randomly between trials and do not predict any particular outcome [6].

To conclude, we demonstrate a positive relationship between outcome-irrelevant learning and compulsive behavior in a healthy volunteer sample. We suggest that attributing value to task representations regardless of their outcome relevance may be one contributory component to the emergence of compulsive behaviors.

## Supplementary information

Supplemental Information
