# Correction: Assigning the right credit to the wrong action: compulsivity in the general population is associated with augmented outcome-irrelevant value-based learning

**DOI:** 10.1038/s41398-021-01734-8

**Published:** 2021-12-02

**Authors:** Nitzan Shahar, Tobias U. Hauser, Rani Moran, Michael Moutoussis, NSPN consortium, Edward Bullmore, Raymond J. Dolan, Ian Goodyer, Peter Fonagy, Peter Jones, Michael Moutoussis, Tobias Hauser, Sharon Neufeld, Rafael Romero-Garcia, Michelle St Clair, Petra Vértes, Kirstie Whitaker, Becky Inkster, Gita Prabhu, Cinly Ooi, Umar Toseeb, Barry Widmer, Junaid Bhatti, Laura Villis, Ayesha Alrumaithi, Sarah Birt, Aislinn Bowler, Kalia Cleridou, Hina Dadabhoy, Emma Davies, Ashlyn Firkins, Sian Granville, Elizabeth Harding, Alexandra Hopkins, Daniel Isaacs, Janchai King, Danae Kokorikou, Christina Maurice, Cleo McIntosh, Jessica Memarzia, Harriet Mills, Ciara O’Donnell, Sara Pantaleone, Jenny Scott, Beatrice Kiddle, Ela Polek, Pasco Fearon, John Suckling, Anne-Laura van Harmelen, Rogier Kievit, Sam Chamberlain, Edward T. Bullmore, Raymond J. Dolan

**Affiliations:** 1 Max Planck University College London Centre for Computational Psychiatry and Ageing Research, London, WC1B 5EH UK; 2 Wellcome Centre for Human Neuroimaging, University College London, London, WC1N 3BG UK; 3 Sagol School of Neuroscience, Tel Aviv University, Tel Aviv, Israel; 4 Psychology Department, Tel Aviv University, Tel Aviv, Israel; 5 Department of Psychiatry, University of Cambridge, Cambridge, UK; 6 Behavioural and Clinical Neuroscience Institute, University of Cambridge, Cambridge, UK; 7 Research Department of Clinical, Educational and Health Psychology, University College London, London, UK; 8 Medical Research Council Cognition and Brain Sciences Unit, University of Cambridge, Cambridge, UK

**Keywords:** Learning and memory, Human behaviour

Correction to: *Translational Psychiatry* 10.1038/s41398-021-01642-x, published online 5 November 2021

The original version of this article unfortunately contained a few minor mistakes in the references generated during an automated proofing process and a typo in Figure 1 (instead of PADWA it should say PADUA). We apologize for the errors. The original article has been corrected.

