# Supplementary material for: Assigning the right credit to the wrong action: compulsivity in the general population is associated with augmented outcome-irrelevant value-based learning
Source: Transl Psychiatry. 2021 Nov 5;11:564. doi: 10.1038/s41398-021-01642-x (PMC8571313; doi:10.1038/s41398-021-01642-x)
Supplement: Supplementary file 1 — Supplemental Information [file 41398_2021_1642_MOESM1_ESM.docx]

**Supplementary Information**

Title: Assigning the right credit to the wrong action: Compulsivity in the general population is associated with augmented outcome-irrelevant value-based learning

Authors: Nitzan Shahar^1,2,3,4^, Tobias U. Hauser^1,2^, Rani Moran^1,2^, Michael Moutoussis^1,2^, NSPN consortium, Edward T Bullmore^5^, Raymond J. Dolan^1,2^

Affiliations

^1^ Max Planck University College London Centre for Computational Psychiatry and Ageing Research, London WC1B 5EH, UK

^2^ Wellcome Centre for Human Neuroimaging, University College London, London WC1N 3BG, United Kingdom

^3^ Sagol School of Neuroscience, Tel Aviv University, Tel Aviv, Israel

^4^ Psychology Department, Tel Aviv University, Tel Aviv, Israel

^5^ Department of Psychiatry, University of Cambridge, Cambridge, UK

* Correspondence to: Nitzan Shahar (PhD)

Psychology Department
Sagol School of Neuroscience
Tel Aviv University
Tel Aviv, Israel

Email: [nitzansh@tauex.tau.ac.il](mailto:nitzansh@tauex.tau.ac.il)

**Contents**

[Materials and methods 3](#_Toc75854384)

[Psychiatric diagnostic exclusion criteria. 3](#_Toc75854385)

[Dimension reduction for self-report measures. 3](#_Toc75854386)

[Reinforcement learning estimates 3](#_Toc75854387)

[Task description. 3](#_Toc75854388)

[Two-step task exclusion criteria 4](#_Toc75854389)

[Oral instructions 4](#_Toc75854390)

[Computational Modeling 4](#_Toc75854391)

[Controlling for age, gender and repeated-assessment across measures. 6](#_Toc75854392)

[Data and code availability. 6](#_Toc75854393)

[Results 7](#_Toc75854394)

[Bayesian correlational and regression analysis. 7](#_Toc75854395)

[Analysis of a single time point 7](#_Toc75854396)

[The influence of instructions on outcome-irrelevant learning 7](#_Toc75854397)

[Controlling for choice-accuracy in first-stage model-based control estimates. 8](#_Toc75854398)

[Repeating the analysis with non-hierarchical measures 8](#_Toc75854399)

[Model-free estimates. 9](#_Toc75854400)

[Tables 10](#_Toc75854401)

[TableS1. Sample characteristics and descriptive data per time-point 10](#_Toc75854402)

[Figures 14](#_Toc75854403)

[References 20](#_Toc75854404)

# Materials and methods

## **Psychiatric diagnostic exclusion criteria**.

The SCID-V was administrated to participants by trained students supervised by a licensed psychiatrist at baseline and the second follow-up sessions. A total of 31 individuals met psychiatric disorders at least at one-time point, including; 9 with major depression disorder, 1 with panic disorder, 2 with social phobia, 6 with specific phobia, 3 with obsessive-compulsive disorder, 2 with post-traumatic stress disorder, 4 with generalized anxiety disorder, 1 with hypochondriasis, 3 with attention-deficit/hyper-activity disorder, 2 with oppositional defiant disorder and 1 with conduct disorder. All 31 individuals were excluded from further analysis.

Dimension reduction for self-report measures.

After obtaining the 25 self-report estimates per individuals (see Table S1), we performed a principal component analysis (pca) using ‘principle’ function included in R ‘psych’ package. This was done following Gillian et al., (2019)^1^ in order to have a better differentiation between obsessions (which was found in Gillian et al., 2019^1^ to load more on anxiety-worry-depression scales) and compulsive behavior, and also reduce the number of sub-clinical estimates for later use. Note that we used we used pca with a promax rotation^2^ rather than factor analysis, as was done by Gillian et al., (2019)^1^. The reason is that pca promax rotation allows obtaining correlated components, while also avoiding a known issue of factor-indeterminacy. The ‘principle’ function in R follows a conventional promax routine, that starts with an orthogonal rotation of the component matrix, then raising the loadings to the power of four^2^ (kappa=4) to produce an ideal factor matrix. Signs of loadings are then restored and the un-rotated component matrix is rotated to the best least squares fit to the ideal factor matrix. This approach is known to allow the extraction of a simplest structure with the lowest correlations among factors.

Following Gillan et al.,^1^ this allowed us to differentiate compulsivity from other psychiatric symptoms, including obsessional thinking which was mostly loaded on the first factor, along with depression, anxiety and worry subscales. To determine the number of factors (or components) we performed a parallel analysis (using 'nfactors function from R's psych package) where we compared the scree of factors from the observed data to factor taken from a random data matrix of the same size as the original (see Figure S6). Specifically, we extracted three factors, which explained 58% of the variance in self-report sub-scales (see Figure 1). The first factor, ‘anxiety-depression-obsession’, explained 22% of the variance and it mostly loaded on sub-scales of worry (loading=.99, RCMAS), depression (loading=.95, MFQ), physiological anxiety (loading=.93, RCMAS), social anxiety (loading=.92, RCMAS), and obsessions (loading=.67/.52, PI-WSUR /OCIR). The second factor, ‘behavioral compulsivity’, explained 21% of variance and loaded on subscales of washing (loading=.91/.91 PI-WSUR /OCIR), ordering (loading=.82, OCI-R), grooming (loading=.83, PI-WSUR), and checking (loading=.74/.70, PI-WSUR /OCI-R). The third factor explained 16% of the variance and was labeled ‘schizotypal tendency’ as it loaded on sub-scales related to having constricted affect (loading=.94, SPQ), no close friends (loading=.93, SPQ), excessive social anxiety (loading=.77, SPQ), and odd speech (loading=.60, SPQ). The three factors model showed good fit to the data with root mean square of the residuals of 0.06, and off-diagonal fit of 0.98. Correlation between the factors are reported in Figure S5.

Reinforcement learning estimates.

Task description.
The task was similar to the one developed by Daw at el., 2011^3^. Participants were instructed to win as much reward (play pounds) as possible, and were told they would receive a payment bonus based on task performance. On each of the stages, subjects had to select one of two fractals. Each trial started by offering two first-stage fractals that appear at the bottom-left and bottom-right sides of the screen. Immediately after making a first-stage choice, the selected fractal appeared in a middle-upper position on the screen for 1.5 sec. The fractals offered during the second-stage were then added to the bottom-left and bottom-right sides of the screen, allowing participants to indicate their second-stage choice. Following the second-stage choice the second-stage selected fractal remained on the screen and a reward (gold coin), or no reward (red ‘X’ mark), appeared in center-bottom side of the screen for 1.5 seconds. The location of the fractals at each state (right vs. left) was randomly selected by the computer at each trial and stage. Participants were instructed to indicate their fractal choice by pressing a right/left arrow on a standard computer keyboard corresponding to the position where the chosen fractal was presented. Instructions noted that response-keys should be used to indicate fractal selection, and that fractals predict reward. Second-stage fractal predicted reward according to a random walk. We counterbalanced between participants two predefined random-walks with (probability range .2 to .8). Both the first and second-stage choice had a 2 seconds response deadline. If the 2 seconds deadline was exceeded ‘Too late. No money earned.’ was presented on screen for 1.5 seconds. Finally, each trial had an inter-trial interval that was randomly selected from a uniformed distribution ranging from 1 to 2 second. The task included 121 trials at Lab-1 and Lab-2 assessments, and 201 trials at Lab-3 (a shorted version in Lab 1 and 2 was given due to time constrained). A short break was provided after half of the trials. 50 practice trials where provided before the test phase. Practice and test phase included a different set of fractal images.

Two-step task exclusion criteria.
To provide a more reliable data set for the two-step task, we administrated pre-processing routines to behavioral data ^4,5^. We excluded participants that had either: (a) more than 10% of the trial data missing due to technical malfunction (two at the first session), (b) more than 10% of the trials missing due to non-responsiveness (two at the first session and one at the third session), (c) responded at the first-stage with the same response-key on more than 95% of the trials (three at the first session), or (d) had implausible reaction-times (below 150ms) on more than 20% of the trials (seven at the first session). For the remaining behavioral data, the first trial in each block, as well as trials with implausible RTs (below 150ms) were omitted from the analysis (~1% of the overall trials).

Oral instructions.
Instruction screen noted: “Welcome to the ‘Two step’ Task! In this task you will have to make two decisions (at two steps) during each trial. At the first step you will choose between two pictures. Each of these will lead you to another pair of pictures. Each of the pictures of the second step can lead you to a reward (a play-pound), or to nothing (an ‘X’). Some of the pictures in the second step USUALLY lead to reward, some only SOMETIMES. Use the LEFT ARROW and RIGHT ARROW keys to make your choices. Your task is to find out which is the best picture and get to it, in order to win points. Note, however, that ‘the best’ pictures will change every now and then, so you will have to keep checking! You will start with some practice rounds to get used to the task. The practice trials will not count towards your score. Feel free to ask the experimenter if anything is unclear at any point”.

Computational Modeling.
We fitted a reinforcement learning model to two-step task’s choice behavior. The model dismantles outcome-relevant learning (model-based) and outcome-irrelevant learning (spatial-motor domain) contribution to participants' choice behavior, and was shown to provide a best fit compared with other models where outcome-irrelevant learning was not integrated^6^. The model uses a temporal difference learning algorithm (TD) wherein predicted values for each choice (represented by Q-values) are updated according to a prediction error teaching signal. The model holds Q^Fractal ­^values (for the six fractals), to Q^key^ values (for the two possible response-keys, right or left), and two Q^MB^ for first-stage fractals, calculated each trial according to transition probability and the subjective expected value of the second-stage fractals^3^.

**Q-values updating:** The six stimulus Q-values were initialized to zero at the beginning of the experiment and updated at the end of each trial. For first-stage choice stimulus values were updated according to:

[1] Q^Stimulus^_(f1,n+1)_=Q^Fractal^ _(f1,n)_ +α_outcome-relevant_ (Q^Fractal^ _(f2,n)_-Q^Fractal^ _(f1,n)_)+ α_outcome-relevant_λ_1_(r_(n)_-Q^Fractal^ _(f2,n)_)

and for second-stage choices according to:

[2] Q^Fractal^_(f2,n+1)_=Q^Fractal^ _(f2,n)_ +α_outcome-relevant_ (r_(n)_-Q^Fractal^ _(f2,n)_)

where f_1_/f_2_ represent selected fractals at the first/second stage, respectively, r_(n)_ represents reward at trial *n* where *r_(n)_ϵ*{0,1}, α_outcome-relevant_ is a fractal learning rate (free parameter) and λ_1_ is an eligibility trace (free parameter) capturing the effect of the second-stage prediction error on first-stage fractal value. Response-key Q values were updated each for the first-stage according to:

[3] Q^Key^_(k1,n+1)_=Q^Key^_(k1,n)_ + α_outcome-irrelevant_*·* λ_2_ (r_(n)_-Q^Key^_(k1,n)_)

and at the second-stage according to:

[4] Q^Key^_(k2,n+1)_=Q^Key^_(k2,n)_ +α_outcome-irrelevant_*·* (1-λ_2_ )(r_(n)_-Q^Key^_(k2,n)_)

where k1/k2 are the response-keys selected for the first and second-stage, accordingly, α_outcome-irrelevant_ is a response-key learning rate (free parameter) and λ_2_ was a free parameter allowing differentiation between credit assignment to the first and second actions when they differ. Model-based learning strategy incorporated the empirical transition probabilities and second-stage Q^Fractal^ values to estimate the value of first-stage actions for each Fractal F according to:

[5] Q^MB^_(F,n)_=P(s_2_|F)*max(Q^Fractal^_(s2,n)_) + P(s_3_|F)*max(Q^Fractal^_(s3,n)_)

where s2/s3 represented the two states in the second stage, and P(s2|F)/ P(s3|F) the transition probability.

**Integrating Q-values:** We then calculated an integrated Q-value for each fractal F, with a w-parameter (*w_1_)* quantified model-based vs. model-free trade-off in first-stage actions^3^ and an additional w-parameter quantifying the contribution of spatial-motor outcome-irrelevant learning^6^. Qnet for the first-stage was calculated according to:

[6] Q^net^_(F,n)_= *w*_model-based_*·*Q^MB^_(F,n)_ + (1-*w*_model-based_)*·*Q^Fractal^_(F,n)_ + *w*_outcome-irrelevant_*·*Q^Key^_(K,n)_

and in the second-stage according to:

[7] Q^net^_(F,n)_= Q^Fractal^_(F,n)_ + *w*_outcome-irrelevant_ *·*Q^Key^_(K,n)_

where K is the response key for selecting fractal F.

**Calculating choice probability:** To the integrated Q-value (Qnet) we added a choice bias value which was an integrated value for three components: (1) fractal choice perseveration (tendency to repeat fractal selection regardless of reward, for first-stage only following previous studies^3^), (2) response-key perseveration (tendency to repeat response-key selection regardless of reward), and (3) response-key bias (reflecting a tendency to use one response-key more than the other, due for example to hand dominancy effects):

[8] *bias*_(a,n)_= *p*_1_*·*Stay^Fractal^_(n)_ + *p*_2_*·*Stay^Response-key^_(n)_*+p*_3_*·*Key_(n)_

*bias*_(a,n)_ describes the amount of bias for a certain action (a) at trial *n*. Here, Stay^Fractal^*ϵ*{0,1} indicated whether the fractal was not selected (0) or selected (1) at the previous trial. Stay^Response-key^*ϵ*{0,1} indicated whether the current response-key was not selected (0) or selected (1) in the previous response. For first-stage choices, Stay^Response-key^ was based on the response-key selected at the second-stage of the previous trial, and for the second-stage choices Stay^Response-key^ was based on the response-key selected at the first-stage of the same trial. Finally, Key_(n)_*ϵ*{0,1} indicated a left (0) or right (1) response-key selection. *p*_1_, *p*_2_ and *p*_3_ are free-parameters that could obtain either negative or positive values. Thus *p*_1_ accounts for a tendency to switch or repeat the previous fractal selection regardless of reward. *p_2_* accounts for a tendency to switch or repeat the previous response-key selection regardless of reward. *p_3_* accounts for a general tendency to select the left or right response-key. The probability of a first/second action was determined using a softmax, with a 1/β parameter representing the decision temperature:

$\left[ 9 \right] P\left( a,t \right)=\frac{\exp\left( \beta[Q^{net}\left( a,n \right)+{bias}\left( a,n \right)] \right)}{\sum_{a'} exp(\beta[Q^{net}\left( a',n \right)+bias\left( a',n \right)])}$

Model fitting. To obtain hierarchical fit we used expectation-maximisation with Laplace approximation method ^7^. In this approach, individual-participant parameters are treated as independent random effects sampled from Gaussian-population distributions (one distribution per parameter), whose means and variances are estimated. For model fitting we collapse all three time-points, searching for a single set of parameters that best predicted all available data (Note that Q-values were reset to zero at the first trial of each time-point measurement).

## **Controlling for age, gender and repeated-assessment across measures**.

All self-report and task-based estimates were controlled for age, gender and repeated-assessment using mixed-effects regression. This allowed us to estimate a single, trait-like score for each individual and each measurement across time-points. We specifically chose to look at estimates beyond time and development since task-based estimates used here are known to be noisy, and combining them across repeated measures was found to greatly improve estimates reliability^4,8,9^. Note that even if true change did accord between time-points in our measurements, our analysis would still be sensitive to explore relationships between latent components (i.e., model-based, outcome-irrelevant learning and compulsivity), since if such a relationship does exist, true change will co-occur between these latent components. However, to assert our results, we repeated the same analysis with data obtained at a single time point (see 'analysis of a single time point' in this document), showing similar results with weaker effects (as should be expected when less indicators are used). Mixed-effect regression was performed using R ‘lme4’ package^10^ for each estimate separately, with age, gender and repeated- assessment (i.e., whether it was the first, second, or third measurement for that participant) as fixed effects, and a random effect of subjects on the intercept. We then used the individual random effect coefficient of the intercept as an estimate for each individual on each estimate.

Data and code availability.

All raw data, processed data and analysis code is available online at the following link <https://osf.io/6h52f/?view_only=6c2cd95f1fab41018a7572114a7bbd12>
DOI 10.17605/OSF.IO/6H52F

To facilitate readers ability to reproduce the analysis, we summaries here the different available code components included in an OSF repository, allowing to reconstruct the full analysis from start to end:
- *‘00_prepro_tst.R’* including the preprocessing sequence for two-step data.
- *‘01_create_subject_list.R’* where participants are excluded based on available measures and SCID data.
- *‘02 create raw data files.R’* where task-based and self-report sub-scores are calculated.
- *‘03_create data matrix.R’* where age, gender and repeated-assessment are controlled for.
- *‘04_main analysis.R’* where PCA is performed.
- ‘ *‘reg.jasp’* JASP file where correlational and linear regression analysis are performed.
- ‘*RL_model.m*’ file with the computational model used to calculate model parameters.

Finally, a folder with all raw and analyzed data is provided. For any questions please contact the first author at nitzansh@tauex.tau.ac.il.

# Results

Bayesian correlational and regression analysis.

Bayesian correlational and regression analyses were implemented using ‘JASP’ software^11^. Bayes factor for Pearson correlations were calculated using a beta-distribution prior centered around zero with a width parameter of 1/3, corresponding to a 80% prior that the correlation coefficient lies between -.5 and +.5. Bayesian regression analysis was performed with a uniform model prior distribution, corresponding to an equal prior for all models, and a parameter prior using JASP default Jeffreys-Zellner-Siow (JZS) prior of 1/8.

**Prior robustness tests.** Bayesian statistics can be sensitive to the selection of priors. Consequently, to demonstrate our results are not due to a specific selection of priors we carried out robustness checks. First, we performed a robustness test for the correlation between outcome-irrelevant learning, model-based control and compulsivity as reported in the main text. We found similar results across a large range of priors (see Figure S3). Second, we followed recent JASP recommendations and tested a wide and ultra-wide parameter JZS priors (1/4 and 1/2) for the Bayesian regressions reported in the main text. We found that overall the regression results did not change in a meaningful way across these set of priors.

Analysis of a single time point.

In the main analysis we aggregated data across multiple-time points to gain a more powerful analysis. Here, we repeat the same analysis with data obtained only from the third in-lab session (lab 3) where we had data collected for all estimates at the same moment (see Table 1 and S1). We included only individuals who had data on all measurements, resulting in 414 participants (mean age=20.37 (sd=2.97), 210 male/204 female). Since aggregating multiple indicators (as done in the main analysis) is known to increase reliability and statistical power, we expected the current analysis (done with only a single time point) to show similar results with weaker effects. We calculated individual scores on the three factor ('anxiety-depression-obsession', 'compulsivity' and 'schizotypal tendencies'), by using the R 'predict' function, using the PCA model from the main text. We re-fitted our computational model to the current set, and calculated model-agnostic scores for model-based control, and outcome-irrelevant learning. We found compulsivity to be negatively related to model-based control (r=-.17, 95%_CI_=-.07 to -.26, BF_10_=49.66), and positively related to outcome-irrelevant learning (r=.12, 95%_CI_=.03 to .21, BF_10_=2.68). Therefore, we found similar evidence in favor of a correlation between compulsivity, model-based and outcome-irrelevant learning to the one reported in the main text, yet in a single time point analysis.

## The influence of instructions on outcome-irrelevant learning

As a response to a reviewer’s comment, we wanted to ensure the effects of outcome-irrelevant learning and its correlation with compulsivity was not due to a misunderstanding, or mistrust in a task's instruction. For this, we performed two analyses:

**Outcome-irrelevant learning in the 'magic carpet' two-step task version.** To explore whether outcome-irrelevant learning might reflect a misunderstanding of the task instructions, we repeated the outcome-irrelevant analysis using an already reported data set that used a story-like instructions^12^. Specifically, Silva and Hare (2020) demonstrated that when the instruction where given in an easy to understand story version, participants showed primarily model-based behavior. We re-analyzed their data (with permission) to examine whether an instruction manipulation eliminated outcome-irrelevant response-key effects in the second-stage (where we previously found these effects to be most pronounced). Specifically, we used a logistic regression where reward in trial n, predict key-repetition trial n+1, only for trials where the state in trial n and n+1 were different (eliminating the influence of credit assignment in the previous trial to the outcome-relevant visual stimuli). The regression further included a random effect of participants on the intercept and slope of the previous-outcome effect. Despite a relatively small sample-size (N=24), we found a statistically significant effect so that participants were more likely to repeat a response-key that was previously followed by reward, then when it was followed by an unrewarded outcome (50.46% vs 51.09% key-repetition on average for previously unrewarded vs. rewarded trials, p<.05). This result shows that outcome-irrelevant learning is not eliminated even when a story-like version of the instruction is given.

**Practice effects.** If we assume that some individuals in our data did not fully understand the instruction, then this should decrease with practice when an individual has had a chance to observe that indeed a response-key does not predict reward. Shahar et al., 2019 showed that in the current data, practice did not reduce outcome-irrelevant learning, and if anything after three sessions of practice in the same task these effects were slightly increased. However, the current study focused on assessing an association of outcome-irrelevant learning to compulsivity and it is possible that some of shared variance is due to individual differences in understanding task instructions. We calculated the number of trials each individual completed and then performed a linear regression where outcome-irrelevant learning, the number of sessions completed (1, 2 or 3) across the whole study, and their interaction predicted compulsivity. We found no change in the association between outcome-irrelevant learning and compulsivity (standardized coefficient for the main effect of outcome-irrelevant on compulsivity was β=0.16, p<.001; main effect of completed sessions was β=-.06, p=.16; interaction β=.03, p=.38). This illustrates that an association of outcome-irrelevant learning and compulsivity did not change when individuals completed more sessions (giving them more chance to observe that indeed effectors are outcome-irrelevant).

Controlling for choice-accuracy in first-stage model-based control estimates.

Akam et al., 2015 demonstrated that correlations between action values in the two-step task can result in an individual who is completely model-free showing an inflated model-based estimate when this is calculated using first-stage choices. Specifically, a completely model-free agent can show a small but significant previous-outcome x previous-transition interaction effect on first-stage choice stay-probability, a common measure of model-based abilities. We follow Akam et al., 2015 recommendations for addressing this issue, and include a choice-accuracy predictor within the regression predicting first-stage choice stay probability, together with previous-reward and previous-transition. Choice accuracy was coded as 1 when the individual chose in the previous trial an option which led commonly to a second-stage state with higher true reward probability, and 0 for choosing option which led commonly to second-stage state with lower true reward probability. When we repeated the same regression analysis reported in the main text using this corrected first-stage model-based score we found similar results. Specifically, we repeated the regression analysis, predicting compulsivity scores using outcome-irrelevant learning, model-based control and their interaction. We found that the best model was one with two main effects and no interaction. This model was 5672 times more likely compared to the null model, 47 more likely compared with a model that had only outcome-irrelevant learning predicting compulsivity, 2.33 more likely compared to a model that had model-based control alone as a predictor for compulsivity and twice more likely compared with a model that included the interaction term. Posterior parameters distributions for the winning model showed that higher outcome-irrelevant learning (coefficient posterior median=0.11, CI_95%_=.02 to .20) and lower model-based abilities (coefficient posterior median=-.16, CI_95%_=-.25 to -.07) predicted higher compulsivity estimates.

## Repeating the analysis with non-hierarchical measures

In the current study we used hierarchical modeling to control for age, gender and repeated measures for each score. Previous studies have noted that the use of hierarchical modeling can exaggerate estimates of covariance and correlations, while underestimating variances^13^. We therefore repeated the same analysis, only this time each score was averaged across repeated measures, without using hierarchical modeling (note we had to omit the computational parameters since these were obtained using hierarchical fitting). Overall, we found that our central finding remained unchanged. We found a positive association between outcome-irrelevant learning and compulsivity (r=0.15, BF10=19.05 in favor of H1). We repeated the linear regression analysis, examining the effect of outcome-irrelevant learning and model-based control on compulsivity. We found that a model with both main effects was 415 more likely given the data compared to a null model (intercept only), 2.15 more likely compared to a model where model-based control was the sole predictor and 14.31 more likely compared to a model with outcome-irrelevant learning as a single predictor. Posterior parameter estimation for the winning model showed a positive effect for outcome-irrelevant learning on compulsivity (coefficient posterior mean=0.11, CI_95%_=.02 to .19) and a negative for the effect of model-based control on compulsivity (coefficient posterior mean=-0.14, CI_95%_=-.22 to -.05). Therefore, our overall central finding was not affected by the use of hierarchical modeling.

Model-free estimates.
The two step-task allows dismantling model-free from model-based estimates using first-stage choices. The model-free system is thought to assign value to first-stage actions based on reward outcomes, regardless of the transition probabilities of the task. Since previous studies failed to show a relationship between model-free estimates and compulsivity, we did not include these in our main analysis, yet included them here for the interested reader. We calculated model-free estimates as the main effect of previous-outcome on stay-probability in first-stage choice. We calculated a Bayesian correlation between model-free estimates and compulsivity and found evidence in favor of the null hypothesis (r= -.07, BF_01_=5.57 in favor of the null), suggesting no association between model-free estimates and compulsivity. Next, we repeated the Bayesian regression analysis reported in the main text with model-free estimates as an additional predictor for compulsivity. We examined the effect of outcome-irrelevant, model-based control and model-free estimates on compulsivity. Overall our conclusion was unchanged with a model that included outcome-irrelevant learning and model-based control as two independent predictors of compulsivity being the most likely one given the data. The winning model was 5.55 times more likely under the data compared with a model that also included model-free as an additional independent predictor. Overall, we did not find evidence for an association between model-free and compulsivity.

# Tables

| TableS1. Sample characteristics and descriptive data per time-point | | | | | | | |
| --- | --- | --- | --- | --- | --- | --- | --- |
|  |  | **HPQ1** | **HPQ2** | **HPQ3** | **Lab1** | **Lab2** | **Lab3** |
| Sample characteristics | N | 514 | 501 | 393 | 514 | 48 | 514 |
|  | Gender (m/f) | 259/255 | 249/252 | 182/211 | 259/255 | 24/24 | 259/255 |
|  | Age | 18.39 (3.01) | 19.57 (3.02) | 20.71 (2.97) | 18.81 (2.96) | 19.30 (2.87) | 20.27 (2.98) |
|  |  |  |  |  |  |  |  |
|  |  |  |  |  |  |  |  |
| LOI | Total score | 4.34 (4.62) | 2.98 (4.11) | 2.61 (3.97) | - | 2.88 (4.41) | 2.8 (3.81) |
|  |  |  |  |  |  |  |  |
| OCI-R | Washing | - | - | - | - | - | .74 (1.69) |
|  | Obsessions | - | - | - | - | - | 1.01 (1.63) |
|  | Hoarding | - | - | - | - | - | 1.77 (1.91) |
|  | Ordering | - | - | - | - | - | 1.74 (2.40) |
|  | Checking | - | - | - | - | - | 1.26 (1.88) |
|  | Naturalizing | - | - | - | - | - | 0.50 (1.27) |
|  |  |  |  |  |  |  |  |
| PI-WSUR | Thoughts about harm | - | 1.71 (2.48) | 1.75 (2.88) | - | - | 1.48 (2.44) |
|  | Impulses to harm | - | 0.83 (1.60) | 0.91 (2.12) | - | - | 0.79 (1.60) |
|  | Contamination | - | 5.08 (6.79) | 4.87 (6.61) | - | - | 4.13 (6.06) |
|  | Checking | - | 5.93 (6.01) | 5.44 (6.51) | - | - | 4.80 (5.63) |
|  | Grooming | - | 0.99 (1.99) | 0.84 (1.76) | - | - | 0.81 (1.81) |
|  |  |  |  |  |  |  |  |
| MFQ | Total | 15.20 (10.21) | 13.43 (10.56) | 12.01 (10.58) | - | 12.78 (10.53) | 12.27 (8.78) |
|  |  |  |  |  |  |  |  |
| RCMAS | Physiological Anxiety | 5.51 (3.86) | 4.55 (3.92) | 4.09 (3.92) |  | 4.51 (5.10) | 4.45 (3.61) |
|  | Worry | 6.61 (5.73) | 5.25 (5.66) | 5.06 (5.85) |  | 4.95 (6.11) | 4.95 (5.17) |
|  | Social concerns | 4.03 (3.43) | 3.25 (3.45) | 2.66 (3.39) |  | 3.12 (3.26) | 2.93 (3.14) |
|  |  |  |  |  |  |  |  |
| SPQ | Ideas of Reference | 2.67 (2.32) | 1.52 (1.92) | 1.18 (1.83) |  | 1.20 (1.49) | 1.34 (1.87) |
|  | Excessive Social Anxiety | 3.26 (2.50) | 2.86 (2.43) | 2.69 (2.52) |  | 2.23 (2.46) | 2.76 (2.39) |
|  | Odd Beliefs | 0.78 (1.19) | 0.40 (0.85) | 0.39 (1.06) |  | 0.54 (1.04) | 0.39 (0.89) |
|  | Unusual Perceptual Experiences | 1.74 (1.84) | 0.93 (1.44) | 0.83 (1.41) |  | 0.83 (1.25) | 0.87 (1.38) |
|  | Odd Behavior | 2 (2.14) | 1.50 (1.99) | 1.27 (1.84) |  | 1.94 (2.36) | 1.29 (1.92) |
|  | No Close Friends | 2.02 (2.05) | 1.69 (2.00) | 1.69 (2.07) |  | 1.51 (1.88) | 1.61 (1.98) |
|  | Odd Speech | 3.12 (2.39) | 2.41 (2.37) | 2.26 (2.32) |  | 2.03 (2.24) | 2.34 (2.31) |
|  | Constricted Affect | 1.79 (1.7) | 1.49 (1.69) | 1.32 (1.60) |  | 1.37 (1.66) | 1.35 (1.63) |
|  | Suspiciousness | 2.27 (2.18) | 1.41 (1.82) | 1.32 (1.83) |  | 1.34 (1.41) | 1.23 (1.67) |
| *Note.* Measurements that were not administrated during a certain time point in the NSPN cohort study are marked with an ‘-‘. HPQ – Home Pack Questionnaire.  *Leyton Obsessional Inventory (*LOI*)* total score has a range from 0 to 33.  *Obsessive-Compulsive Inventory–Revised* (OCI-R) subscales has a range from 0 to 4.  *Padua Inventory-Washington State University Revision* (PI-WSUR) subscales as a range from 0 to 28 (Thoughts about harm), 0 to 36 (Impulses to harm), 0 to 40 (Contamination, Checking), and 0 to 12 (Grooming).  *Mood and Feelings Questionnaire* (MFQ) has a total score ranging from 0 to 99.  *Revised Children’s Manifest Anxiety Scale* (RCMAS) subscales ranged from 0 to 30 (Physiological-anxiety), 0 to 33 (Worry), 0 to 21 (Social-anxiety).  *Schizotypal Personality Questionnaire* (SPQ) subscales ranged from 0 to 9 (Ideas of reference, Odd speech, Magical-thinking, No close friends, Perceptual experiences), 0 to 8 (Excessive social-anxiety, Constricted affect, Suspiciousness), and 0 to 7 (Odd behavior). | | | | | | | |

| **Table S2.** NHT regression analysis with outcome-irrelevant learning and model-based control predicting compulsivity. | | | | | | |
| --- | --- | --- | --- | --- | --- | --- |
|  | | **Predictor** | **Standardized** | | **t** | **p** |
|  |  | Model-based control |  | -0.138 | -3.045 | <.01 |
|  |  | Outcome-irrelevant learning |  | 0.127 | 2.813 | <.01 |
|  | | | | | | |

| **Table S3.** Bayesian regression analysis with outcome-irrelevant learning and model-based control predicting compulsivity, while controlling for depression-anxiety-obsessions, schizotypal factors. | | | | | | | | | | | |
| --- | --- | --- | --- | --- | --- | --- | --- | --- | --- | --- | --- |
| **Models** | |  | | |  | | **BF _10_** | | **R²** | |  |
| outcome-irrelevant learning |  |  |  |  | |  |  | 1.000 |  | 0.396 |  |
| Null model (including. depresion-anxiety-obsessions, schizotypal) |  |  |  |  | |  |  | 0.339 |  | 0.387 |  |
| model-based control |  |  |  |  | |  |  | 0.325 |  | 0.393 |  |
| model-based control + outcome-irrelevant learning |  |  |  |  | |  |  | 0.309 |  | 0.398 |  |
| model-based control + outcome-irrelevant learning + model-based control  ✻  outcome-irrelevant learning |  |  |  |  | |  |  | 0.092 |  | 0.401 |  |
|  | | | | | | | | | | | |
| *Note.* BF _10_ estimates refer to Bayes factor in favor of the winning model (outcome-irrelevant learning as a single predictor). All models include and intercept, depression-anxiety-obsessions, and schizotypal effects om compulsivity as null predictors. | | | | | | | | | | | |

| **Table S4.** NHT regression analysis with outcome-irrelevant learning and model-based control predicting compulsivity, while controlling for depression-anxiety-obsessions, schizotypal factors. | | | | |
| --- | --- | --- | --- | --- |
| **Predictor** | | **Standardized** | **t** | **p** |
|  | Depression-anxiety-obsessions factor | 0.483 | 10.617 | < .001 |
|  | Schizotypal factor | 0.165 | 3.672 | < .001 |
|  | Model-based control | -0.056 | -1.552 | n.s |
|  | Outcome-irrelevant learning | 0.078 | 2.166 | <.05 |
|  | | | | |


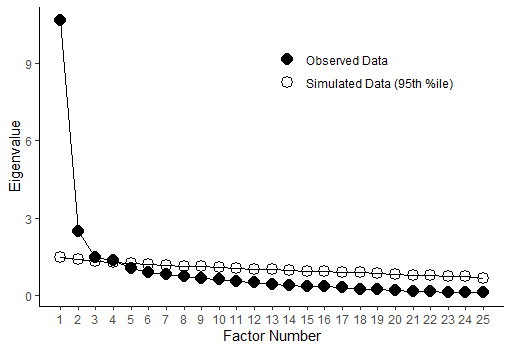


**FigureS**1. Scree plot showing the eigenvalue of the observed data and simulated random data matrix (using R 'nfactor' function from 'psych' package).

# Figures


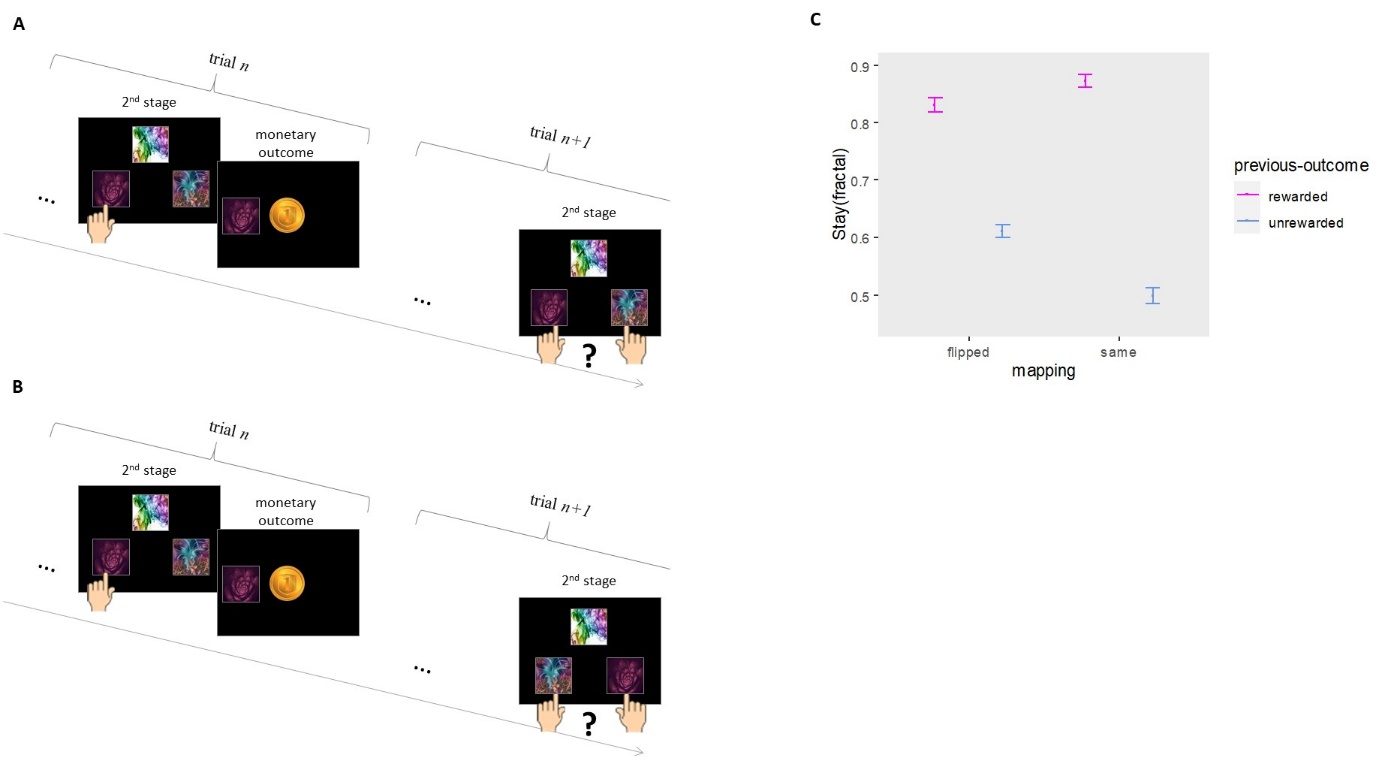


**FigureS2**. Second-stage score II for outcome-irrelevant learning. Here we calculated outcome-irrelevant learning from trials where the same set of fractals was offered in the second-stage on the *n* and *n+1* trial. If some credit is assigned to the response-key (reflecting outcome-irrelevant learning), we should expect a greater previous-outcome effect on trials where the mapping was the *same* compared with when it was *flipped*. When the mapping is the *same* as in the previous trial, both the previously selected fractal and response-key are associated with the same choice in the following trial. Credit assigned to the response-key should therefore enhance the effect of previous-outcome on the probability that the individual will stay with the same fractal selection as the previous trial (i.e., pStay_fractal_). However, on *flipped* trials, the response-key that was previously affiliated with the chosen fractal is now affiliated with the alternative fractal. Therefore, on *flipped* trials, credit-assignment to the response-key should reduce the previous-outcome influence on the probability that the individual will stay with the previous fractal selection (i.e., pStay_fractal_). Panel C shows a previous-outcome x mapping interaction (p<.001) in line with credit assignment to the response-keys.

**FigureS3**. Model-based scores. (A) First-stage model based score was calculated as the interaction between previous-transition and previous-reward on the probability that the individual will stick with the same first-stage choice^3,4,14^. (B) Second-stage model-based score was calculated as the second-stage reaction-time differences after rare vs. common transition^4,15^.


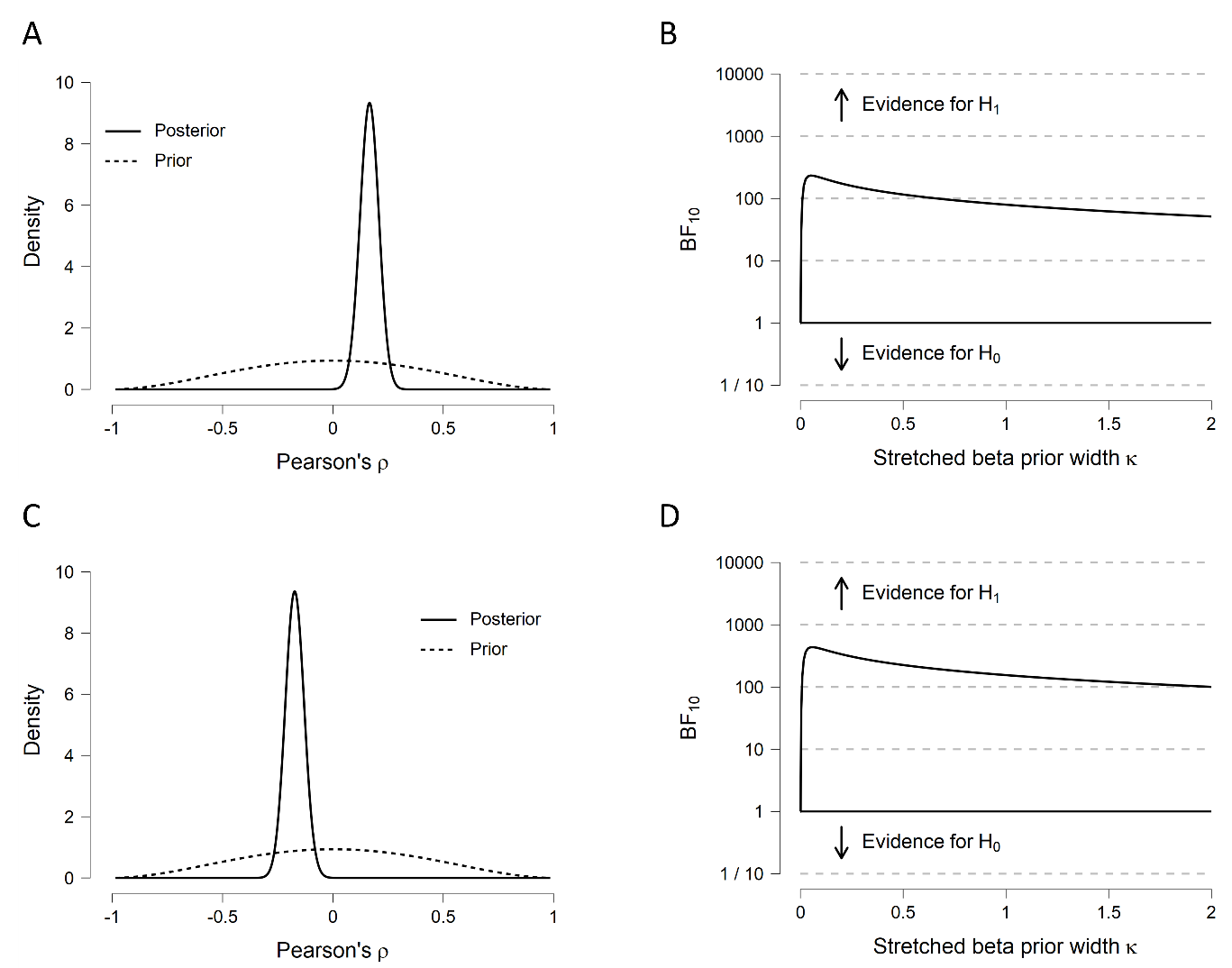


**FigureS4**. Bayesian parameter estimates for the two correlations between outcome-irrelevant learning, and model-based control with compulsivity. (**A**) Posterior and prior distributions (solid and dashed lines, respectively) for the Pearson correlation estimate between outcome-irrelevant learning and compulsivity. (**B**) Priors robustness check demonstrating that evidence in favor of a correlation between outcome-irrelevant learning and compulsivity is robust across a range of priors. (**C**) Posterior and prior distribution (solid and dashed lines, respectively) for a Pearson correlation estimating the association between model-based control and compulsivity. (**D**) Priors robustness check demonstrating that evidence in favor of a correlation between model-based control and compulsivity is robust across a range of priors.


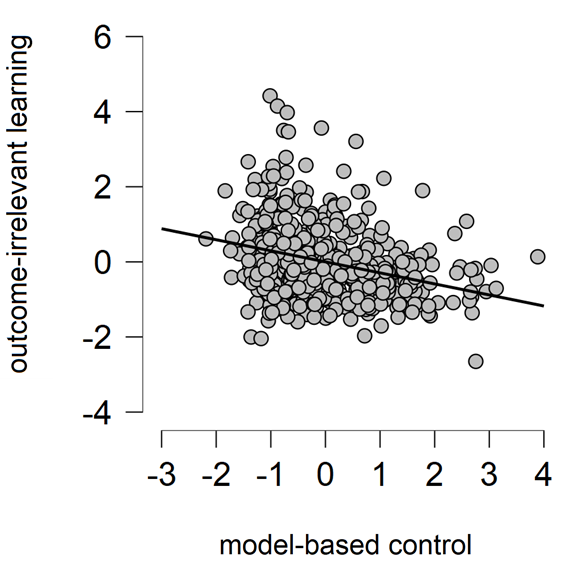


**FigureS5**. Scatter plot for the negative relationship between outcome-irrelevant learning and model-based control.


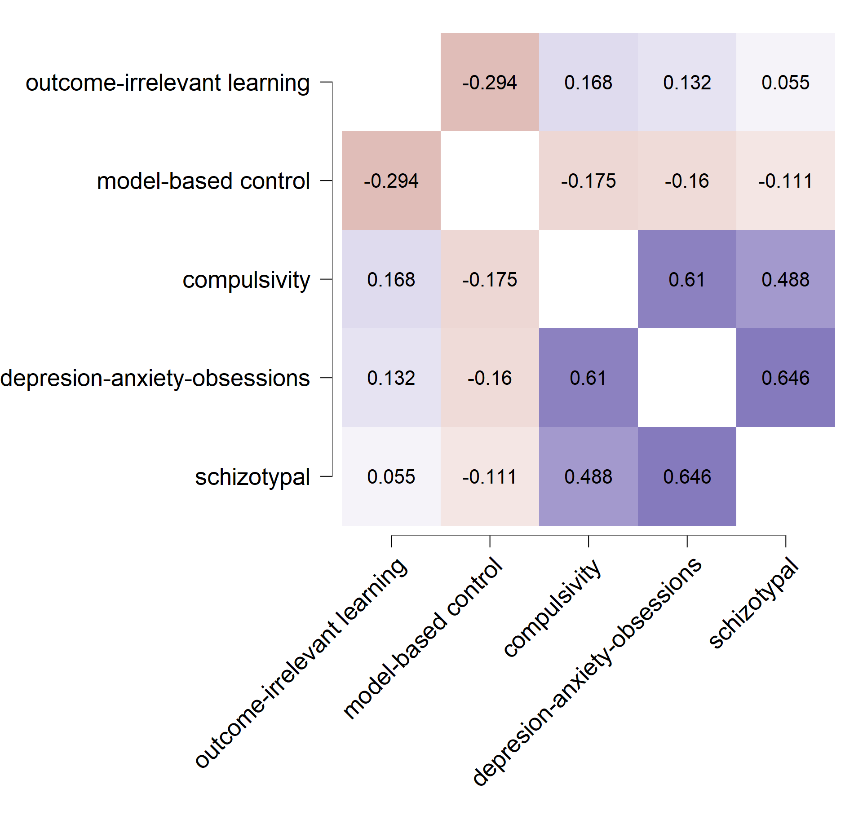


**FigureS6**. Correlation matrix showing the association between the two task-based and three self-report latent factors.

# References

1. Gillan CM, Kalanthroff E, Evans M, et al. Comparison of the Association Between Goal-Directed Planning and Self-reported Compulsivity vs Obsessive-Compulsive Disorder Diagnosis. *JAMA Psychiatry*. Published online October 9, 2019:1-10. doi:10.1001/jamapsychiatry.2019.2998

2. Hendrickson AE, White PO. Promax: A quick method for rotation to oblique simple structure. *Br J Stat Psychol*. 1964;17(1):65-70. doi:10.1111/j.2044-8317.1964.tb00244.x

3. Daw ND, Gershman SJ, Seymour B, Dayan P, Dolan RJ. Model-based influences on humans’ choices and striatal prediction errors. *Neuron*. 2011;69(6):1204-1215. doi:10.1016/j.neuron.2011.02.027

4. Shahar N, et al. Improving the reliability of model-based decision-making estimates in the two-stage decision task with reaction-times and drift-diffusion modeling. *PLOS Comput Biol*. 2019;15(2):e1006803. doi:10.1371/journal.pcbi.1006803

5. Gillan CM, Kosinski M, Whelan R, Phelps EA, Daw ND. Characterizing a psychiatric symptom dimension related to deficits in goal-directed control. *eLife*. 2016;5. doi:10.7554/eLife.11305

6. Shahar N, et al. Credit assignment to state-independent task representations and its relationship with model-based decision making. *Proc Natl Acad Sci*. 2019;116(32):15871-15876. doi:10.1073/pnas.1821647116

7. Huys QJM, et al. Disentangling the Roles of Approach, Activation and Valence in Instrumental and Pavlovian Responding. *PLOS Comput Biol*. 2011;7(4):e1002028. doi:10.1371/journal.pcbi.1002028

8. Kaplan D. *Structural Equation Modeling: Foundations and Extensions*. SAGE Publications; 2008.

9. Enkavi AZ, et al. Large-scale analysis of test–retest reliabilities of self-regulation measures. *Proc Natl Acad Sci*. 2019;116(12):5472-5477. doi:10.1073/pnas.1818430116

10. Bates D, Maechler M, Bolker B, Walker S. Fitting linear mixed-effects models using lme4. *Journal of Statistical Software*. 2015;67(1):1-48. doi:doi:10.18637/jss.v067.i01

11. JASP T. *JASP Team. JASP (Version 0.12.2)[Computer Software].*; 2020.

12. Silva C, Hare TA. Humans primarily use model-based inference in the two-stage task. *Nat Hum Behav*. Published online July 6, 2020:1-14. doi:10.1038/s41562-020-0905-y

13. Kliegl R, Masson MEJ, Richter EM. A linear mixed model analysis of masked repetition priming. *Vis Cogn*. 2010;18(5):655-681. doi:10.1080/13506280902986058

14. Kool W, Cushman FA, Gershman SJ. When does model-based control pay off? *PLoS Comput Biol*. 2016;12(8). doi:10.1371/journal.pcbi.1005090

15. Deserno L, et al. Ventral striatal dopamine reflects behavioral and neural signatures of model-based control during sequential decision making. *Proc Natl Acad Sci U S A*. 2015;112(5):1595-1600. doi:10.1073/pnas.1417219112
